# Investigating the impact of a mutation in PDE5A on myocardial infarction

**DOI:** 10.1186/2050-6511-16-S1-A43

**Published:** 2015-09-02

**Authors:** Tan A Dang, Jana Wobst, Thorsten Kessler, Simon V Ameln, Manuel Lehm, Matthias Prestel, Ingrid Braenne, Redouane Aherrahrou, Martin Dichgans, Christian Hengstenberg, Jeanette Erdmannn, Heribert Schunkert

**Affiliations:** 1 Deutsches Herzzentrum München, Klinik fuer Herz- und Kreislauferkrankungen, Technische Universität Muenchen, Munich, Germany; 2 Institut für Schlaganfall- und Demenzforschung, Ludwig-Maximilians-Universität München, Munich, Germany; 3 Universität zu Lübeck, Institut fuer Integrative und Experimentelle Genomik, Lübeck, Germany; 4 Deutsches Zentrum fuer Herz- und Kreislaufforschung (DZHK) e.V., partner site Munich Heart Alliance (MHA), Munich, Germany; 5 Deutsches Zentrum fuer Herz- und Kreislaufforschung (DZHK) e.V., partner site Hamburg/ Kiel/ Lübeck, Lübeck, Germany

## 

Recently, our group has discovered a family suffering from premature coronary artery disease (CAD) and myocardial infarction (MI). Whole-exome sequencing of three affected family members revealed that these shared a mutation in the PDE5A gene encoding the phosphodiesterase 5A protein. Mutations in proteins involved in cGMP-signalling have already been shown to play an important role in premature CAD/MI [1]. Here, we aimed to characterize the effect of this mutation at the molecular level.

*PDE5A* encodes three distinct isoforms, PDE5A1, PDE5A2, and PDE5A3, all of which cleave cGMP, a second messenger that plays an important role in e.g. smooth muscle relaxation and thrombocyte passivation. The particular mutation is located in the first intron of PDE5A1 and PDE5A3, which is known to be an alternative promoter site [2], and in the first exon of PDE5A2, generating a premature stop codon. Stable isotope labeling by amino acids in cell culture (SILAC) analysis in HeLa S3 nucleus cell lysates revealed allele-specific binding of the transcription factor ZFX to the major allele. In line, reporter gene analysis of the first intron of PDE5A1 and PDE5A3 by luciferase assay pointed to significantly different promoter activities of the investigated alleles in HEK293 cells with 40% increased activity of the mutated allele. Furthermore, overexpression experiments of the mutated construct in HEK293E cells did not show a loss of transcript as supposed by the premature stop codon but hinted towards the expression of an N-terminal truncated PDE5A2 isoform.

We demonstrated an increased promoter activity of the mutated PDE5A allele which might lead to increased expression of PDE5A isoforms. This differential promotor activity could be mediated by allele-specific binding of the transcription factor ZFX, which is part of current studies. As the N-terminus of PDE5A, i.e. the GAF-domain, act as an important regulator of enzymatic activity [3], we hypothesize that the truncated PDE5A2 isoform might be more active. Overall, our results point towards a gain of function mutation in PDE5A associated with premature CAD and MI.
